# Multimorbidity associated with anxiety symptomatology in post-COVID patients

**DOI:** 10.1016/j.psychres.2022.114427

**Published:** 2022-03

**Authors:** Milena Nogueira Azevedo, Elisângela da Silva Rodrigues, Emília Augusta Franz Vieira Passos, Márcio Andrade Barreto Filho, Ana Paula Andrade Barreto, Marcelo Chalhoub Coelho Lima, Mauricio Lima Barreto, Luis Fernando Silva Castro-de-Araujo

**Affiliations:** a Hospital Especializado Octávio Mangabeira (HEOM), Salvador, Brazil; b Universidade Federal da Bahia (UFBA), Salvador, Brazil; c Escola Bahiana de Medicina e Saúde Pública (EBMSP), Salvador, Brazil; d Center of Data and Knowledge Integration for Health (CIDACS); e Faculdade de Tecnologia e Ciências (FTC), Salvador, Brazil; f Universidade Salvador (UNIFACS), Salvador, Brazil; g The University of Melbourne, Victoria, Australia; h Universidade Federal do Ceará, Ceará, Brasil

**Keywords:** COVID-19, long-covid, quality of life

## Abstract

The COVID-19 pandemic hit individuals with chronic conditions the hardest. It is known that anxiety symptoms are frequent in post-COVID conditions. We want to examine whether multimorbidity is associated with anxiety in post-COVID patients. We reported descriptive statistics from 389 post-COVID patients and perform a linear regression with anxiety symptoms measured using the Hospital Anxiety and Depression (HAD) scale. For each extra chronic condition, there was a mean increase of 0.11 in the HAD-anxiety score. However, there was a reduction for age and being male. These findings can potentially help policy-makers better organize post-COVID health services and improve patients care.

## Highlights

• Few studies have described post-covid conditions in a population, mainly de low/middle-income country.
• Multimorbidity was significantly associated with anxiety symptomatology.
• Being male and aging reduced the anxiety and depression score.

## 1 Introduction

The current pandemic has raised concerns regarding the mental health of those affected by social isolation. There have been reports of increased psychiatric symptomatology following social isolation (Borges Machado et al., 2020; Castro-de-Araujo and Machado, 2020), especially among younger subjects (Pieh et al., 2020).

The appearance of neurological and psychiatric symptomatology has also been reported following SARS-COV-2 infection. There are reports of lasting anxiety, depression, and post-traumatic stress symptoms following infection, impacting patients` quality of life (Rogers et al., 2020). Long COVID has been described as including persistent fatigue, headaches and `brain fog`(Stefano et al., 2021). Anxiety symptomatology has recently been reported to occur in up to 31.4% of recovered COVID-19 patients post-hospitalization (Morin et al., 2021).

Multimorbidity is a global health priority (Sciences, T.A.of M. 2018). This pandemic had the greatest effect on individuals with chronic conditions. It is expected that these individuals have concerns regarding their own health and the risks of becoming (re)infected, especially in a country where, arguably, the vaccination roll-out was slower. Describing and analyzing changes in mental state in post-COVID patients who are also multimorbid is fundamental to policy-making and to prevent worse outcomes. Therefore, this paper investigates whether multimorbidity is associated with an increase in anxiety symptoms in a sample of subjects who attend an outpatient service for post-COVID conditions.

## 2 Methods

The sample included 389 patients that attended in the Centro Pós-COVID-19 (CPC), a public health outpatient clinic that operates at the Octávio Mangabeira Specialized Hospital, located in Salvador-Bahia, Brazil. Patients were referred by medical practitioners at COVID treatment centres across the State of Bahia, following local health authority recommendations. The patients were interviewed by a multidisciplinary team that included trained psychologists, pneumologists, nurses and social workers from August 2020 to March 2021. The team members used a structured interview developed for a larger prospective cohort, which aimed to evaluate several clinical and psychological measures in individuals with persistent symptoms following the acute COVID-19 phase. Data were collected and managed using Research Electronic DataCapture (REDCap) software, with hosting provided by the Gonçalo Moniz Institute (IGMFIOCRUZ), located in Bahia, Brazil (Harris et al., 2019, 2009). This study was approved by the institutional review board of the Bahia State University (UNEB; protocol no. 38,281,720.2.0000.0057) and the Santo Antonio Hospital ethics committee (OSID; protocol no. 33,366,030.5.0000.0047).

### 2.1 Outcome

Anxiety symptoms were assessed using the Hospital Anxiety and Depression (HAD) scale (Zigmond and Snaith, 1983), which was previously validated in Portuguese (Botega et al., 1995). HADS-A is a subscale designed to measure anxiety symptoms in community samples. The anxiety score from this scale was used in the model as the outcome.

### 2.2 Independent variables

The independent variables of interest consisted of a count of the clinical conditions which were acquired during the interviews: hypertension, heart disease, diabetes mellitus, chronic obstructive pulmonary disease (COPD), asthma, chronic kidney disease, obesity, and cancer. In order to align with the definition of multimorbidity (reporting a minimum of two chronic conditions), this variable was coded starting at 2, and if the patient only had one condition, this was coded as zero.

### 2.3 Confounders

The following variables were included in the model as a form of adjustment: age, sex, time elapsed since initial COVID-19 symptoms, race (black/mixed race, white, and others) and education level (illiterate, basic reading skills, elementary school, high school, and graduate). Analysis was performed with linear regression adjusted to a t-student distribution, using the gamlss package in the R statistical environment, version 4.0.4. The model passed the normality of residuals assumption (supplementary material).

## 3 Results

The data set consisted of 240 females (61.7%) and 149 males (38.3%), who were mostly black/mixed race subjects (88.7%), with a mean age of 51.82 years (SD 14.04). At the acute COVID-19 phase, 59.6% of patients required hospitalization. The mean score for HADS-anxiety was 8.21 (SD 4.74) and 7.16 (SD 4.73) for HADS-depression. The cognitive performance did not differ significantly between sexes, and the mean Mini-Mental State Examination (MMSE) (Folstein et al., 1975) score was 25.60 (SD 3.38) (Table 1).

**Table 1:** Main demographic characteristics of the sample. Group comparison was performed using Chi-2 tests for the categorical variables and ANOVA for the continuous variables (anxiety, depression scores).

	Overall (N=389)	Females (N=240)	Males (N=149)	P-Value
**Age, years**	51.82 (14.04)	50.38 (13.88)	54.13 (14.03)	0.010
**BMI, kg/m^2^**	29.58 (6.33)	30.01 (7.14)	28.88 (4.67)	0.089
**Skin color**				0.047
Black/Mixed	345 (88.7)	219 (91.2)	126 (84.6)	
White	38 (9.8)	16 (6.7)	22 (14.8)	
Other	5 (1.3)	4 (1.7)	1 (0.7)	
**Educational level**				0.227
Illiterate	4 (1.0)	3 (1.2)	1 (0.7)	
Reading ability	68 (17.5)	36 (15.0)	32 (21.5)	
Elementary school	72 (18.5)	48 (20.0)	24 (16.1)	
High school	164 (42.2)	97 (40.4)	67 (45.0)	
Graduate level	50 (12.9)	37 (15.4)	13 (8.7)	
**Work**				0.047
Employed	118 (33.8)	77 (35.5)	41 (31.1)	
Unemployed	79 (22.6)	56 (25.8)	23 (17.4)	
Others	152 (43.6)	84 (38.7)	68 (51.5)	
**Health worker**	72/383 (18.8)	62/238 (26.1)	10/145 (6.9)	<0.001
**Sedentary**	188/285 (66)	127/179 (70.9)	61/106 (57.5)	0.021
**Alcohol use**				0.004
No	221 (56.8)	146 (60.8)	75 (50.3)	
In social events	149 (38.3)	85 (35.4)	64 (43.0)	
Daily	6 (1.5)	0 (0.0)	6 (4.0)	
**Smoking habits**				0.001
Never smoked	287 (73.8)	189 (78.8)	98 (65.8)	
Ex-smoker	88 (22.6)	39 (16.2)	49 (32.9)	
Currently smokes	6 (1.5)	0 (0.0)	6 (4.0)	
**Time of disease onset, months**	3.06 (1.83)	3.19 (1.92)	2.85 (1.65)	0.072
**Hospitalized**	232/382 (60.7)	117/234 (50.0)	115/147 (78.2)	<0.001
**Days hospitalized**	11.0 (6.0–18.0)	12.0 (7.0–20.0)	11.0 (6.0–18.0)	0.285
**ICU**	121/232 (52.1)	54/117 (46.1)	67/115 (58.2)	0.075
**Multimorbidity**	157/380 (40.7)	98/233 (42.1)	59/147 (40.1)	0.711
**Obesity**	162 (41.6)	107 (44.8)	55 (37.4)	0.155
**Cardiovascular disease**	30/378 (7.9)	19/231(8.2)	11/147 (7.5)	0.366
**Hypertension**	171/380 (45)	102/233 (43.8)	69/147 (46.9)	0.503
**Asthma**	44/379 (11.6)	30/232 (12.9)	14/147 (9.5)	0.290
**COPD**	14/375 (3.7)	8/231 (3.5)	6/144 (4.2)	0.922
**Diabetes mellitus**	78/380 (20.5)	44/233 (18.9)	34/147 (23.1)	0.366
**Chronic renal disease**	2/379 (0.5)	1/232 (0.4)	1/147 (0.7)	0.458
**Cancer**	5/379 (1.3)	3/232 (1.3)	2/147 (1.4)	0.482
**HADS anxiety score**	8.21 (4.74)	9.61 (4.47)	5.83 (4.21)	<0.001
**HADS anxiety classification**	N=313	N=197	N=116	<0.001
Normal	144 (46.0)	61 (31.0)	83 (71.6)	
Borderline Abnormal	67 (21.4)	54 (27.4)	13 (11.2)	
Abnormal	102 (32.6)	82 (41.6)	20 (17.2)	
**HADS depression score**	7.16 (4.72)	8.58 (4.57)	4.75 (3.96)	<0.001
**MMSE**	25.6 (3.38)	25.33 (3.38)	26 (3.35)	0.074
**Sleep disturbance**	273/387 (70.5)	186/238 (78.2)	87/149 (58.4)	<0.001
**Appetite change**	240/385 (62.3)	164/236 (69.5)	76/149 (51)	<0.001
**Previous contact with psychologist**	77/382 (20.2)	62/239 (25.9)	15/143 (10.5)	<0.001
**Previous psychopharmacology use**	97/386 (25.1)	74/237 (31.2)	23/149 (15.4)	<0.001
**Were you refereed to a psychologist?**	137/350 (39.1)	100/211 (47.4)	37/139 (26.6)	<0.001

Data are n (%), n/N (%), mean (SD) or median (IQR)

BMI - Body mass index; COPD - Chronic obstructive pulmonary disease; MMSE - Mini Mental State Examination

Most of the individuals were sedentary (52.9% in females and 40.9% in males), did not smoke (73.8%), or consume alcohol (56.8%). However, there were significant differences among strata, since all of the daily alcohol drinkers were female (4%), and all of the participants who currently smoke were male (1.2%). Thirty percent were formally employed. Eighteen percent were health workers, with significant differences between sexes (25.8% of the women were health workers). The majority of patients had the equivalent of a high school level of education (42.2%), with no difference between sexes.

A number of specific symptoms were surveyed. There were significant differences between sexes in terms of changes in appetite and sleep disturbance (Table 1). Females were more likely to experience sleep disturbance (77.5%) and a change in appetite (68.3%). Previous consultations with a psychologist, a history of psychoactive medication, and having been referred to a psychologist in the past were also more frequently described among females (25.8%, 30.8% and 41.7%, respectively).

The impact of anxiety symptoms was assessed using the HADS-A in 313/389 patients. 169 (54%) reported some degree of alteration (HADS-A ≥8). Female sex (P<0.001) and age (P<0.001) showed a significant correlation with the scale classification (Supplementary table 1). Of the 169 altered patients, 80.4% were women, with 60.3% of then presenting a HADS-A score above 10 (Anxiety Case).

An inferential analysis was performed using linear regression modeling. Our main interest was investigating the association of multimorbidity with anxiety symptoms as the outcome. We found that multimorbidity was significantly associated with anxiety symptomatology, measured using the HADS (Figure 1 - supplementary material). In Figure 1, we report the standardized coefficients, and raw estimates can be found in the supplementary material. For each additional chronic condition, there was a mean increase of 0.11 on the HADS-anxiety score. However, in contrast, there was a reduction for age and being male on the HADS-anxiety score (-0.19 and -0.85 respectively). This model passed the normality of residual assumption, presented an overall good fit, and was the best performing model in a likelihood ratio test (supplementary material).

## 4 Discussion

This is a preliminary analysis from data on a larger cohort of post-COVID patients in a lower middle-income country highly affected by the COVID pandemic. We found that multimorbidity was significantly associated with anxiety symptomatology at the baseline. Each additional chronic condition increased by an average of 0.11 on the score measuring anxiety symptomatology.

Multimorbid patients were one of the first groups to be outlined as being a high mortality risk during the COVID pandemic. This information raised concerns regarding their health status in the post-COVID phase. Multimorbidity was a prevalent condition in our data, and is often associated with a worse health-related quality of life (Prazeres and Santiago, 2016). The effect of multimorbidity on quality of life may be negatively affected by interaction with post-COVID-19 conditions, and a higher risk of subsequent infection with worse outcomes. The requirement to attend follow-up consultations with specialists causes stress, since this could potentially expose them to a second infection.

In addition to multimorbidity, our study highlights that being female and younger were also associated with a worse HADS-anxiety score. A previous preprint study of our group also found that women's quality of life was more affected, and they attained worse scores in the anxiety/depression domain of the EuroQoL questionnaire (EQ-5D-5 L) (Andrade Barreto et al., 2021). Female predominance in post-COVID symptomatology and a poorer quality of life have been discussed in previous studies (Huang et al., 2021). Another Brazilian study evaluating the adherence of multimorbid patients to preventive COVID-19 measures, found that females presented a higher prevalence of multimorbidity, and better adherence to social isolation, in comparison to males (Batista et al., 2020).

Our findings should be interpreted with caution. The data set is small, and the sample consisted of subjects who were referred to a post-COVID treatment center in a state capital, so it may not be generalizable to the general population. Furthermore, this is a cross-sectional study and, therefore, no causal relationships can be inferred.

Despite these limitations, this study is the first to report a significant association between anxiety symptoms and the number of chronic conditions in post-COVID patients in a Brazilian sample. If confirmed in larger samples, and over time, this study has implications for primary care physicians, confirming the need for an early evaluation of mental health symptoms (anxiety and depression in particular) in multimorbid post-COVID patients, in order to prevent worse outcomes. This may also assist policymakers to better allocate resources, thereby preventing potential mental disorders and worsening chronic clinical conditions. It essential that a further evaluation and better description of the clinical course of anxiety symptomatology is performed in the future, and specific, higher risk populations are analyzed (women, those with low-incomes, and health professionals).

## Author statement

APAB, MLB, MCCL,MNA conceived, designed, and supervised the study. ESR e LFSCA performed the formal analysis. APAB, MABF, MNA, EAFVP were responsible for data management. MABF, APAB, LFSCA, ESR, EAFVP, MNA interpreted the data, and wrote the first draft. MLB, MCCL critically revised the manuscript for important intellectual content. The corresponding author attests that all listed authors meet authorship criteria and that no others meeting the criteria have been omitted.

## Declaration of Competing Interest

Authors report no conflicts of interest.

## Appendix Supplementary materials

Image, application 1
