# Supplementary material for: Multimorbidity associated with anxiety symptomatology in post-COVID patients
Source: Psychiatry Res. 2022 Mar;309:114427. doi: 10.1016/j.psychres.2022.114427 (PMC8801057; doi:10.1016/j.psychres.2022.114427)
Supplement: Supplementary file 1 [file mmc1.docx]

# **SUPPLEMENTAL MATERIAL**

**Supplementary Figure 1 - Standardized coefficients of a linear model fitted to the t-distribution. MM = multimorbidity**


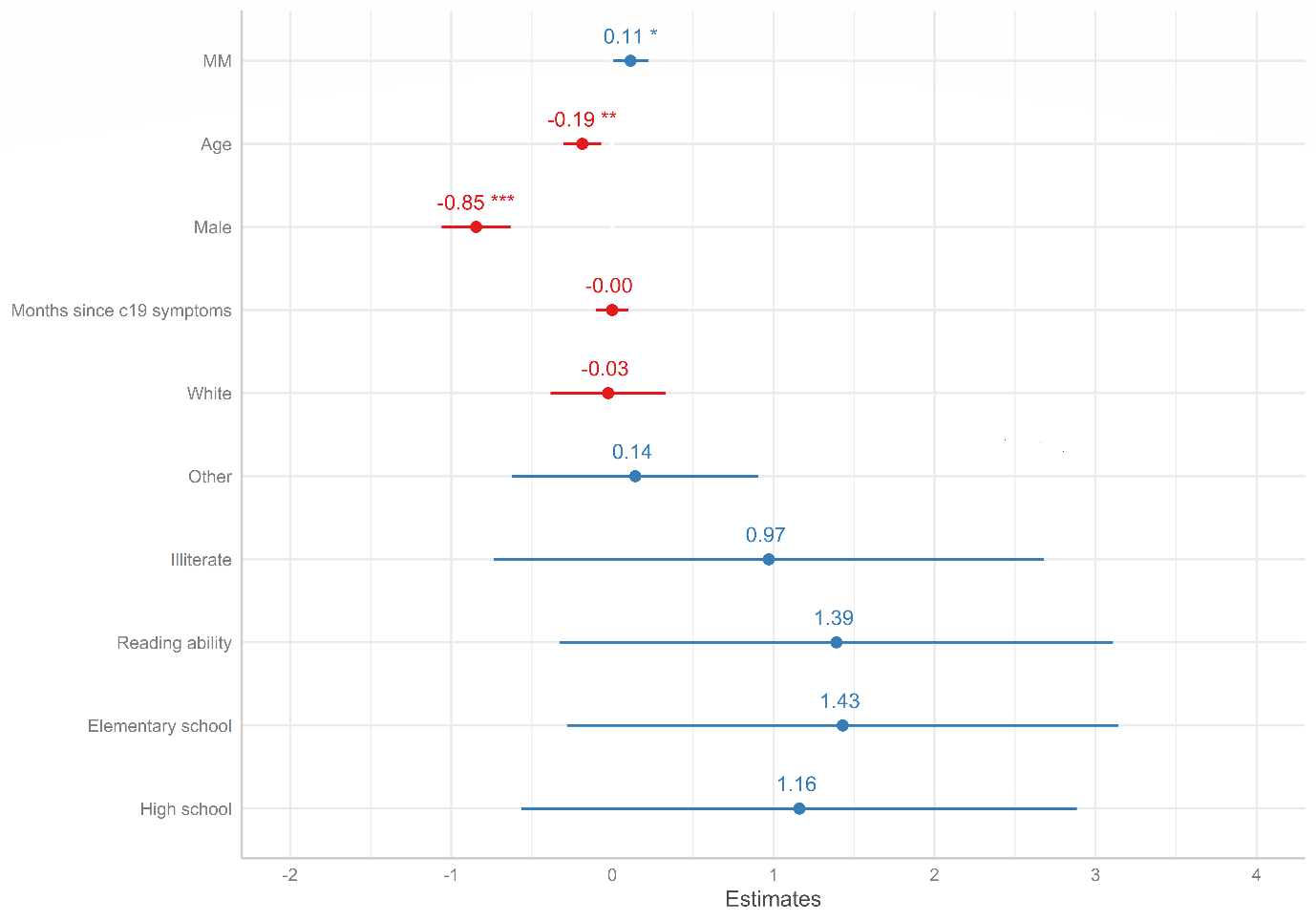


##

## **Supplementary Table1. HADS-A classification**

| **Variable** | **HADS ≤7**  **N=144** | **HADS 8-10**  **N=67** | **HADS ≥11**  **N=102** | **P-Value** |
| --- | --- | --- | --- | --- |
| **Female** | 61 (42.4) | 54 (80.6) | 82 (80.4) | P<0.001 |
| **Age** | 54.4 (±13.4) | 49.5 (±12.6) | 45.5 (±12.1) | P<0.001 |
| **Race** |  |  |  | 0.293 |
| Black-mixed | 125 (86.8) | 60 (89.6) | 94 (92.2) |  |
| White | 18 (12.5) | 5 (7.5) | 6 (5.9) |  |
| Others | 1 (0.7.0) | 2 (3.0) | 2 (2.0) |  |
| **BMI** | 29.1 (±5.9) | 30.3 (±5.7) | 30.7 (±7.9) | 0.089 |
| **Hospital admission** | 88/139 (63.3) | 38/66 (57.6) | 54/101 (53.5) | 0.302 |
| **Hypertension** | 66/138 (47.5) | 26/66 (39.4) | 38/101 (37.6) | 0.242 |
| **DM** | 27/138 (19.6) | 14/66 (21.2) | 13/101 (12.9) | 0.286 |
| **Obesity** | 59/141 (41.8) | 32 (47.8) | 51 (50.0) | 0.424 |
| **CODP** | 5/135 (3.6) | 1/66 (1.5) | 4/100 (4.0) | 0.646 |
| **Asthma** | 9/138 (6.5) | 12/66 (18.2) | 15/100 (15.0) | 0.027 |
| **Cancer** | 1/138 (0.7) | 0/66 (0.0) | 0/100 (0.0) | 0.547 |
| **Hearth disease** | 15/138 (10,9) | 2/66 (3) | 4/99 (4.0) | 0.046 |
| **DRC** | 1/139 (0.7) | 0/66 (0.0) | 0/101 (1.0) | 0.547 |
| Data are n (%), n/N (%), mean (±SD) or median (IQR). Group comparison was performed using Chi-2 tests for the categorical variables and ANOVA/ kruskal-wallis for the continuous variables.  BMI - body mass index; COPD - chronic obstructive pulmonary disease. | | | | |

## **Supplementary Table 2. Model estimates**

|  | **Estimate** | **Std. Error** | **t value** | **Pr(>\|t\|)** |
| --- | --- | --- | --- | --- |
| (Intercept) | 6.51 | 4.35 | 1.49 | 0.14 |
| MM | 0.43 | 0.21 | 2.01 | 0.04 |
| age | -0.07 | 0.02 | -3.10 | 0.00 |
| sexMale | -4.00 | 0.52 | -7.76 | 0.00 |
| months_since_c19_sympt | 0.00 | 0.13 | -0.03 | 0.98 |
| colorWhite | -0.12 | 0.86 | -0.14 | 0.89 |
| colorOther | 0.68 | 1.85 | 0.37 | 0.72 |
| scholarityIlliterate | 4.60 | 4.12 | 1.12 | 0.27 |
| scholarityReadingAbility | 6.58 | 4.15 | 1.59 | 0.11 |
| scholarityHighSchool | 6.77 | 4.13 | 1.64 | 0.10 |
| scholarityGraduate | 5.50 | 4.16 | 1.32 | 0.19 |

##

## **Supplementary Table 3. Likelihood ratio test**

| Models | LRT | df | P-value |
| --- | --- | --- | --- |
| m1-m2 | 0.036 | 1 | 0.85 |
| m2-m3 | 0.16 | 2 | 0.92 |
| m3-m4 | 12 | 4 | 0.016 |

- m1 = HAD-A ~ MM + age + sex

- m2 = HAD-A ~ MM + age + sex + months_since_c19_sympt

- m3 = HAD-A ~ MM + age + sex + months_since_c19_sympt + colour

- m4 = HAD-A ~ MM + age + sex + months_since_c19_sympt + colour + scholarity

### **Normality of the residuals**

Shapiro-Wilk normality test

data: object$residuals

W = 1, p-value = 0.02

One-sample Kolmogorov-Smirnov test

data: object$residuals

D = 0.05, p-value = 0.5

alternative hypothesis: two-sided

### **Measures of goodness-of-fit**

**
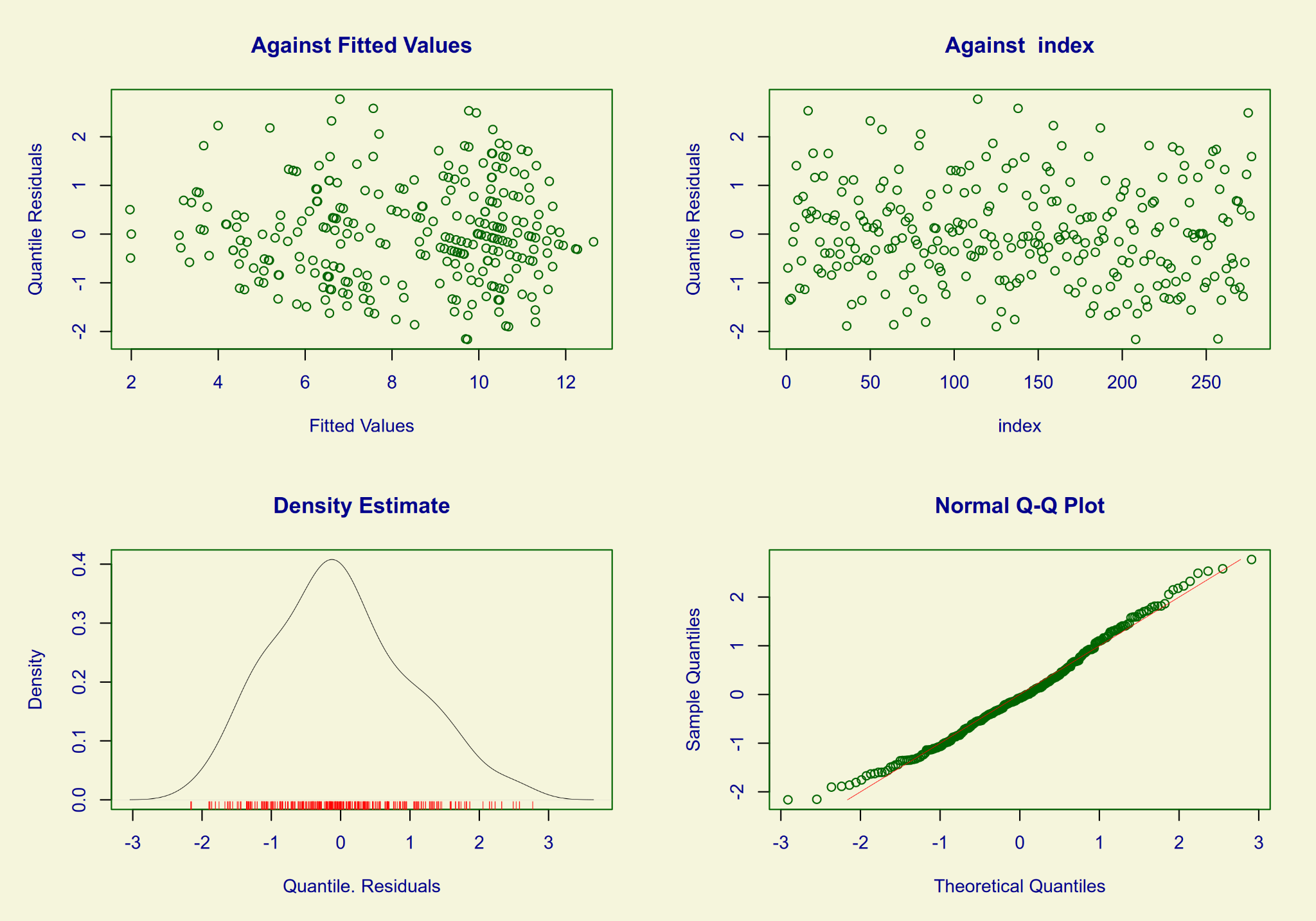
**

******************************************************************

Summary of the Quantile Residuals

mean = 0.00000000000000087

variance = 1

coef. of skewness = 0.33

coef. of kurtosis = 2.7

Filliben correlation coefficient = 0.99

******************************************************************

##

**Missing data patterns**

**
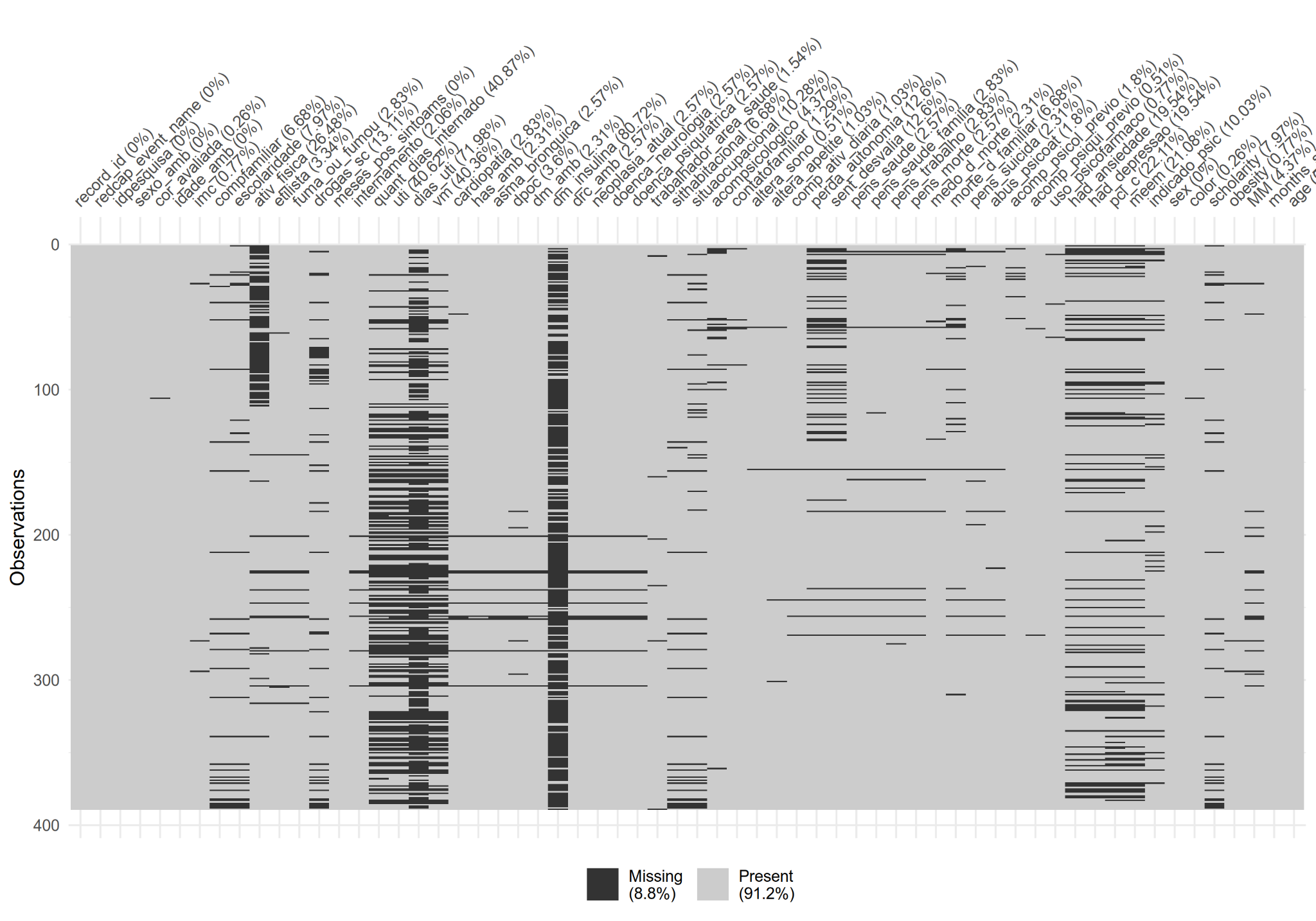
**

###

## **System information**

R version 4.0.4 (2021-02-15)

Platform: x86_64-solus-linux-gnu (64-bit)

Running under: Solus 4.2 Fortitude

Matrix products: default

BLAS/LAPACK: /usr/lib64/haswell/libopenblas_haswellp-r0.3.13.so

locale:

[1] LC_CTYPE=en_US.UTF-8 LC_NUMERIC=C LC_TIME=pt_BR.UTF-8 LC_COLLATE=en_US.UTF-8

[5] LC_MONETARY=pt_BR.UTF-8 LC_MESSAGES=en_US.UTF-8 LC_PAPER=pt_BR.UTF-8 LC_NAME=C

[9] LC_ADDRESS=C LC_TELEPHONE=C LC_MEASUREMENT=pt_BR.UTF-8 LC_IDENTIFICATION=C

attached base packages:

[1] parallel splines stats graphics grDevices utils datasets methods base

other attached packages:

[1] gamlss_5.2-0 nlme_3.1-152 gamlss.dist_5.1-7 MASS_7.3-53 gamlss.data_5.1-4

[6] sjPlot_2.8.8 GGally_2.1.1 oddsratio_2.0.1 ggsci_2.9 margins_0.3.26

[11] kableExtra_1.3.4 naniar_0.6.0.9000 visdat_0.5.3 tableone_0.12.0 rmarkdown_2.7

[16] summarytools_0.9.9 forcats_0.5.1 stringr_1.4.0 dplyr_1.0.4 purrr_0.3.4

[21] readr_1.4.0 tidyr_1.1.2 tibble_3.1.0 ggplot2_3.3.3 tidyverse_1.3.0

[26] here_1.0.1 ProjectTemplate_0.10.1 nvimcom_0.9-82

loaded via a namespace (and not attached):

[1] readxl_1.3.1 backports_1.2.1 systemfonts_1.0.1 plyr_1.8.6 TH.data_1.0-10 pryr_0.1.4

[7] digest_0.6.27 htmltools_0.5.1.1 magick_2.7.1 fansi_0.4.2 magrittr_2.0.1 checkmate_2.0.0

[13] modelr_0.1.8 matrixStats_0.58.0 sandwich_3.0-0 svglite_2.0.0 colorspace_2.0-0 rvest_0.3.6

[19] mitools_2.4 haven_2.3.1 xfun_0.21 tcltk_4.0.4 crayon_1.4.1 jsonlite_1.7.2

[25] lme4_1.1-26 survival_3.2-7 zoo_1.8-8 glue_1.4.2 gtable_0.3.0 emmeans_1.6.1

[31] webshot_0.5.2 UpSetR_1.4.0 sjstats_0.18.1 sjmisc_2.8.7 rapportools_1.0 scales_1.1.1

[37] mvtnorm_1.1-1 DBI_1.1.1 ggeffects_1.1.0 Rcpp_1.0.6 viridisLite_0.3.0 xtable_1.8-4

[43] performance_0.7.2 proxy_0.4-25 prediction_0.3.14 survey_4.0 httr_1.4.2 RColorBrewer_1.1-2

[49] ellipsis_0.3.1 pkgconfig_2.0.3 reshape_0.8.8 farver_2.1.0 dbplyr_2.1.0 utf8_1.1.4

[55] tidyselect_1.1.0 labeling_0.4.2 rlang_0.4.10 effectsize_0.4.5 munsell_0.5.0 cellranger_1.1.0

[61] tools_4.0.4 cli_2.3.1 generics_0.1.0 sjlabelled_1.1.8 broom_0.7.5 evaluate_0.14

[67] yaml_2.2.1 knitr_1.31 fs_1.5.0 pander_0.6.3 xml2_1.3.2 compiler_4.0.4

[73] rstudioapi_0.13 e1071_1.7-6 reprex_1.0.0 statmod_1.4.35 stringi_1.5.3 highr_0.8

[79] ps_1.6.0 parameters_0.14.0 lattice_0.20-41 Matrix_1.3-2 nloptr_1.2.2.2 vctrs_0.3.6

[85] pillar_1.5.0 lifecycle_1.0.0 estimability_1.3 data.table_1.14.0 insight_0.14.1 R6_2.5.0

[91] gridExtra_2.3 codetools_0.2-18 boot_1.3-26 assertthat_0.2.1 rprojroot_2.0.2 withr_2.4.1

[97] multcomp_1.4-16 mgcv_1.8-33 bayestestR_0.10.0 hms_1.0.0 grid_4.0.4 labelled_2.7.0

[103] coda_0.19-4 class_7.3-18 minqa_1.2.4 snakecase_0.11.0 lubridate_1.7.10 base64enc_0.1-3
